# Yeokwisan: Standardised Herbal Formula Enhancing Gastric Mucosal Protection Against Gastric Ulcers in Mice, a Preclinical Study

**DOI:** 10.3390/ph18010044

**Published:** 2025-01-02

**Authors:** Yun Mi Lee, Kyuhyung Jo, So Yeon Kim, Chang-Seob Seo, Eunjung Son, Aejin Kim, Dong-Seon Kim

**Affiliations:** 1 KM Science Research Division, Korea Institute of Oriental Medicine, Daejeon 34054, Republic of Koreacsseo0914@kiom.re.kr (C.-S.S.);; 2 KM Convergence Research Division, Korea Institute of Oriental Medicine, 1672 Yuseong-daero, Yuseong-gu, Daejeon 34054, Republic of Korea; 3 Korean Convergence Medical Science Major, Campus of Korea Institute of Oriental Medicine, University of Science & Technology, 1672 Yuseong-daero, Yuseong-gu, Daejeon 34054, Republic of Korea

**Keywords:** Yeokwisan, gastritis, alcohol, restraint stress

## Abstract

**Background**: Yeokwisan (YWS) is a standardised herbal formula for relieving functional dyspepsia symptoms. **Methods**: We explored the therapeutic value of YWS and its potential effects on gastritis. Its inhibitory effect on gastric mucosal damage and anti-inflammatory activity in animal models of alcohol- and restraint stress-induced gastritis were also examined. Gastric tissues of ICR mice treated with YWS (150 and 300 mg/kg) or famotidine (5 mg/kg) for 10 days were collected, and gastric lesions were quantified. The stomachs of C57BL/6 mice treated with YWS (150 and 300 mg/kg) or famotidine (5 mg/kg) for 23 days were collected, and gastric lesions were quantified. Blood samples were analysed for inflammation related factors and gastroprotective effects. **Results**: YWS (300 mg/kg) inhibited gastric damage by 42.33% in the EtOH-induced gastritis model and 75% in the restraint stress-induced gastritis model (compared to the control group). Pretreatment with YWS led to decreased levels of inflammatory factors (IL-1β, IL-6, and COX-2). YWS showed gastroprotective effects through histamine downregulation, while prostaglandin E2 (PGE2) and mucin were upregulated. The mRNA levels of H2R, M3R, CCK2R, and H^+^/K^+^ ATPase were significantly decreased following treatment with YWS. **Conclusions:** YWS provides gastric protection through its anti-inflammatory properties, reduced histamine secretion, and enhanced release of mucosal defensive factors.

## 1. Introduction

Gastritis is a gastrointestinal disorder characterised by irritation, inflammation, and erosion of the gastric epithelium. It is generally influenced by various factors, including alcohol consumption, stress, dietary habits, smoking, the use of nonsteroidal anti-inflammatory drugs (NSAIDs), and *Helicobacter pylori* infection [1,2]. The pathophysiology of gastritis is determined by the imbalance between aggressive factors, such as gastric acid and pepsin, and defensive factors, including mucosal blood flow and prostaglandin E2 (PGE2) [3,4]. The treatment of gastric ulcers includes acid-suppressing medications, such as histamine-2 receptor antagonists (H_2_ blockers) and proton pump inhibitors (PPIs), which reduce gastric acid secretion and enhance mucosal protection. However, the use of these medications is limited due to gastrointestinal toxicity and other underlying causes [5,6]. Long-term PPI use can cause various structural and functional mucosal changes associated with the development of gastric cancer, particularly under conditions of *H. pylori* infection [7]. Furthermore, the US Food and Drug Administration recalled ranitidine (an H2 receptor antagonist) medications owing to concerns over potential cancer-causing impurities [8]. Consequently, finding effective treatment protocols to mitigate these side effects is essential. Herbal medicine, i.e., plant-derived treatments, has been used to treat many diseases. Recently, plant extracts and isolated phytochemicals have been reported as potential drugs for the treatment of gastritis, being associated with no toxicological risks even with long-term chronic exposure [9]. Numerous studies have reported on the significant therapeutic effects of various classical formulations, proprietary herbal medicines, and active ingredients of herbal medicine in pharmacological experiments and clinical applications. In addition, they have been revealed to have pharmacological effects through mechanisms such as anti-inflammatory, anti-apoptotic, and antioxidant, gastric mucosal protection, analgesic, immune modulation, and anti-emetic actions [10,11]. Yeokwisan (YWS) is a standardised herbal medicine consisting of six medicinal herbs, i.e., *Scutellaria baicalensis* Georgi, Massa medicata Fermentata, *Ostrea gigas* Thunberg, *Poncirus trifoliata* Rafinesque, *Phyllostachys bambusoides* Sieb. et Zucc., and *Glycyrrhiza uralensis* Fischer. YWS is clinically prescribed for patients with refractory functional dyspepsia (FD) and gastroesophageal reflux disease (GERD). Studies on the effects and the mechanisms of action of this medicine have been reported in a murine model of loperamide-induced FD [10]. In our previous study, 13 compounds were simultaneously quantified in YWS samples, and the antioxidant effects of these compounds were reported [12]. Here, we quantified 14 compounds by the addition of neoponsyrin and explored the therapeutic effects and potential mechanisms of action in a gastrointestinal disease model to determine the possibility of expanding the indications of YWS, which is used for FD with symptoms similar to gastritis and GERD with similar therapeutic drugs. Therefore, in this study, we investigated the inhibitory effects of YWS on gastric mucosal damage and its anti-inflammatory activity in gastric tissue using animal models of alcohol- and restraint stress-induced gastritis. Additionally, a chemical analysis of YWS was performed using high-performance liquid chromatography (HPLC).

## 2. Results

### 2.1. HPLC-PDA Profiling Analysis of YWS

**Figure 1:**
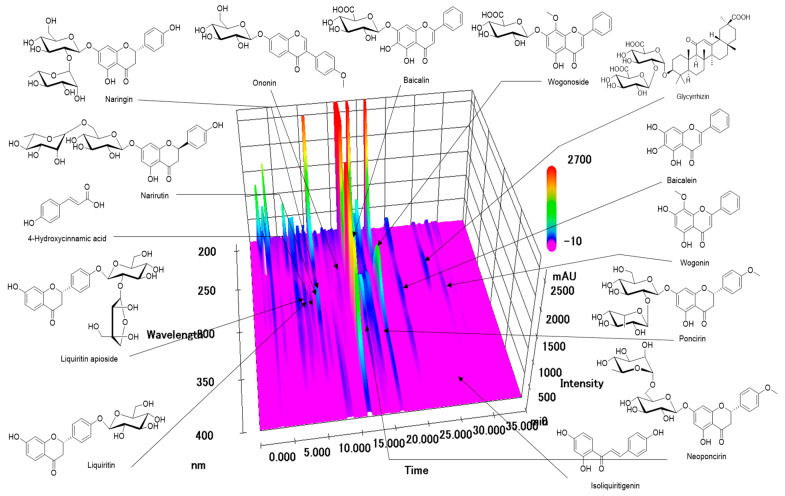
Three-dimensional HPLC chromatograms of freeze-dried Yeokwisan (YWS) sample.

Phytochemical profiling and quantification of the selected 14 components from the YWS sample were performed using the established HPLC-PDA simultaneous analysis method. The 14 components were quantified using PDA at the following wavelengths based on the maximum ultraviolet absorption wavelength of the standard compound (Table 1): 250 nm (ononin and glycyrrhizin), 275 nm (liquiritin apioside, liquiritin, baicalin, wogonoside, baicalein, and wogonin), 280 nm (narirutin, naringin, neoponcirin, and poncirin), 310 nm (4-hydroxycinnamic acid), and 370 nm (isoliquiritgenin). These target components were completely eluted within 35 min, and the three-dimensional HPLC chromatogram is shown in Figure 1. In the YWS sample, 14 target components were detected at concentrations of 0.03 to 89.45 mg/freeze-dried g. In particular, baicalin (a main compound in *S. baicalensis*) was the highest at 89.45 mg/freeze-dried g.

### 2.2. Effect of Gastroprotective and Antiulcer Activities

#### 2.2.1. Model of Ethanol-Induced Gastritis

**Figure 2:**
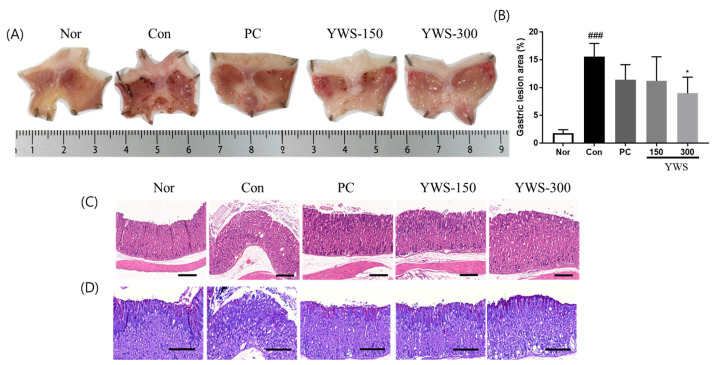
Gastroprotective effect of YWS against ethanol-induced ulceration in mice. (**A**) Representative images of murine stomachs. (**B**) Pathological injury index. Histological analysis of (**C**) haematoxylin and eosin (H&E) and (**D**) periodic acid-Schiff (PAS) staining in gastric tissue. Scale bar, 200 µm. Data are expressed as means ± standard error of the mean (SEM) (n = 5). ^###^ *p* < 0.001 versus the normal group (Nor); and * *p* < 0.05 versus the control group (Con).

The effects of YWS on gastric lesions are shown in Figure 2. In the gastritis control group (Con), EtOH administration caused severe gastritis in animals treated with only 0.5% CMC, characterised by erosion and haemorrhage, with an affected area of 15.58 ± 7.54%. YWS (300 mg/kg) significantly inhibited gastric lesion formation by 42.33% compared to the gastritis control group (*p* < 0.001).

#### 2.2.2. Model of Restraint Stress-Induced Gastritis

**Figure 3:**
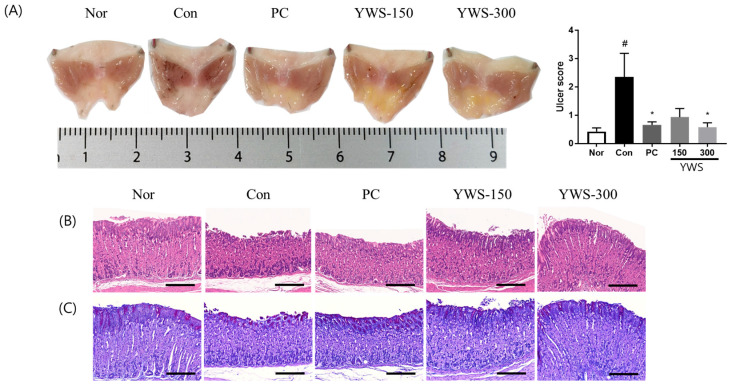
Effect of YWS on gastric injury in mice with restraint stress-induced gastric ulcers (**A**). Data are expressed as means ± standard error of the mean (SEM) (n = 8). Histological analysis of (**B**) haematoxylin and eosin (H&E) and (**C**) periodic acid–Schiff (PAS) staining in gastric tissue. # *p* < 0.05 versus the non-stressed group (Nor); and * *p* < 0.05 versus the stressed group (Con). Scale bar, 200 µm.

In animals subjected to restraint stress, structural histopathological changes, such as gastric haemorrhagic lesions and epithelial cell damage, were observed. Conversely, treatment with YWS (300 mg/kg) significantly alleviated total gastric mucosal damage by reducing the UI score by 75% compared to the stressed control group (Con). The standard drug used in this experiment, famotidine (PC, 5 mg/kg), reduced gastric damage by 71.72% (Figure 3).

#### 2.2.3. Histopathological Examination

The groups treated with famotidine and YWS exhibited reduced cellular loss and mucosal tissue damage compared to the control group. As depicted in Figure 2 and Figure 3, both EtOH and stress-reduced mucus secretion, as evidenced by a decrease in the intensity of magenta staining in the gastric tissue. However, the famotidine and YWS-treated groups exhibited high magenta intensities, indicating the presence of mucosal barriers.

### 2.3. Effect of YWS on Serum Gastrin, Corticosterone, Histamine, and PGE2 Levels in a Model of Restraint Stress-Induced Gastritis

**Figure 4:**
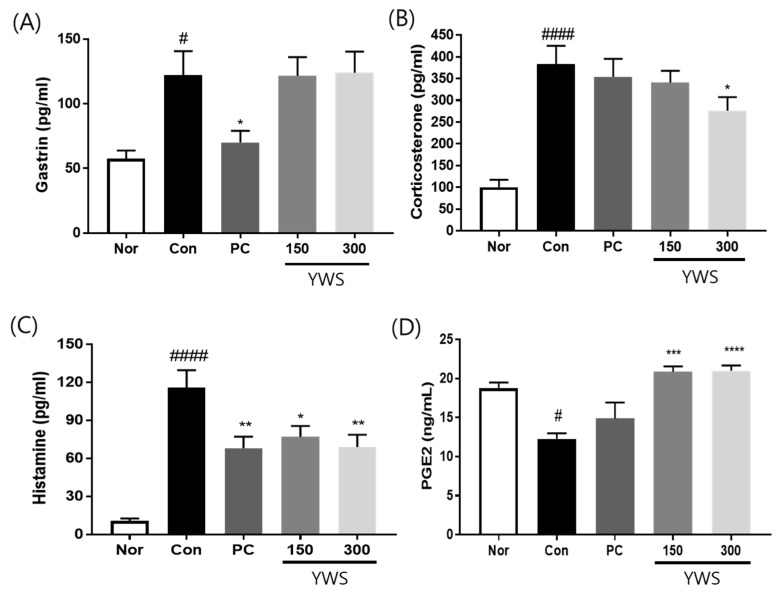
Effect of YWS on stress-induced gastrin, corticosterone, histamine, and PGE2 levels. YWS affected the accumulation of (**A**) gastrin, (**B**) corticosterone, (**C**) histamine, and (**D**) PGE2 levels. Data are expressed as the mean ± SEM (n = 8). ^#^ *p* < 0.05 and ^####^ *p* < 0.0001 versus the non-stressed group (Nor); and * *p* < 0.05, ** *p* < 0.01, *** *p* < 0.001, and **** *p* < 0.0001 versus the stressed group (Con).

Figure 4 shows that exposure to restraint stress significantly increased the levels of gastrin, corticosterone, and histamine in the stress-exposed group (Con) (compared to the unstressed group (Nor)). In contrast, in the positive control group treated with famotidine (PC), gastrin levels decreased significantly; however, YWS (150 and 300 mg/kg) did not affect the decrease in gastrin levels (Figure 4A). Nonetheless, treatment with 300 mg/kg YWS significantly reduced corticosterone and histamine production by 27.88% and 40.30%, respectively (Figure 4B,C). PGE2 levels were significantly lower in the stress-exposed group than in the unstressed group, and pretreatment with 150 and 300 mg/kg of YWS substantially increased PGE2 levels (Figure 4D).

### 2.4. Effect of YWS on the mRNA Expression of Gastric Acid Secretion-Related Factors and Mucin

**Figure 5:**
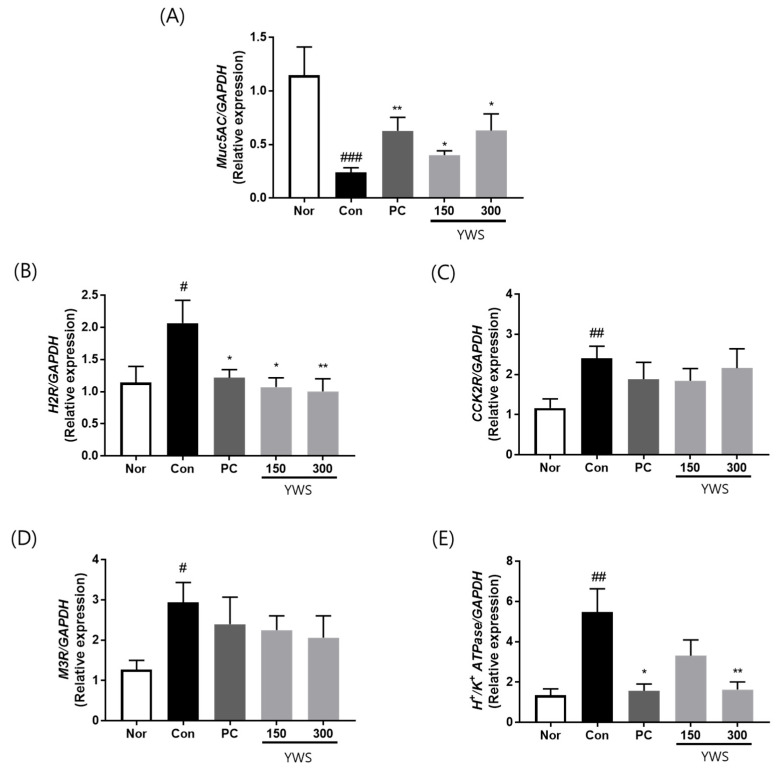
Effect of YWS on the expression of genes associated with gastric mucin and factors promoting gastric acid secretion in a model of restraint stress-induced gastritis. mRNA levels of (**A**) MUC5AC, (**B**) histamine H2-receptors (H2R), (**C**) cholecystokinin-2/gastrin receptors (CCK2R), (**D**) muscarinic acetylcholine receptor M3 receptor (M3R), and (**E**) H^+^/K^+^ ATPase in the gastric mucosa. Data are expressed as the mean ± SEM (n = 8). ^#^ *p* < 0.05, ^##^ *p* < 0.01, and ^###^ *p* < 0.001 versus the non-stressed group (Nor); * *p* < 0.05, and ** *p* < 0.01, versus the stressed group (Con).

To confirm that the gastroprotective effects of YWS were associated with receptor activation and mucin expression, gene expression levels were measured using qRT-PCR (Figure 5). The results showed that the mRNA expression of muscarinic receptor (M3R), histamine receptor (H2R), cholecystokinin-2 receptor (CCK2R), and H^+^/K^+^ ATPase was significantly higher in the stress-exposed group (Con) than in the unstressed group (Nor). However, H2R and H^+^/K^+^ ATPase were significantly downregulated upon YWS pretreatment at 300 mg/kg, whereas M3R and CCK2R showed a non-significant decreasing trend. These results suggest that YWS inhibits the mRNA expression of precursor acid secretion factors, such as H2R, M3R, and CCK2R, leading to a decrease in H^+^/K^+^ ATPase. Furthermore, the decreased expression of MUC5AC, a major protective component of the gastric mucosa under stress, significantly increased in the YWS-treated (150 and 300 mg/kg) and PC groups.

### 2.5. Effect of YWS on cAMP Concentrations in the Gastric Mucosa

**Figure 6:**
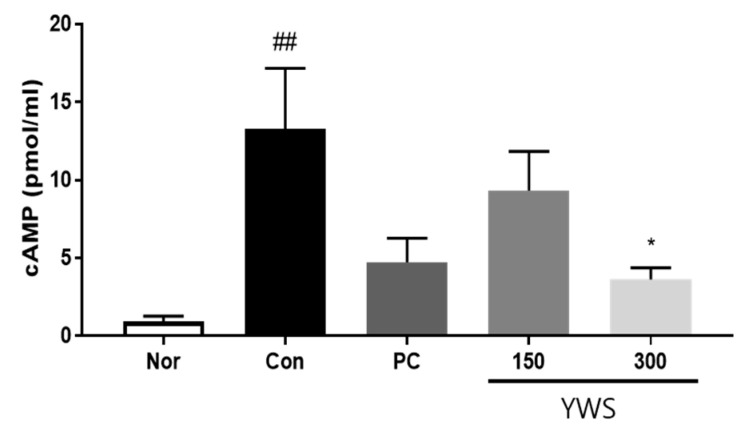
Effect of YWS on stress-induced cAMP levels. YWS reduced the accumulation of cAMP. Data are expressed as the mean ± SEM (n = 8). ^##^ *p* < 0.01 versus the non-stressed group (Nor) and * *p* < 0.05 versus the stressed group (Con).

cAMP levels were measured to investigate whether YWS is associated with gastric acid secretion via cAMP in the gastric mucosa. In the model of restraint stress-induced gastritis, YWS reduced gastric cAMP levels (Figure 6). In particular, treatment with 300 mg/kg YWS reduced cAMP activity to a degree similar to that observed upon using the H2R antagonist famotidine.

### 2.6. Effect of YWS on the Expression of Pro-Inflammatory Cytokines and COX-2 in a Model of EtOH-Induced Gastric Ulcer

**Figure 7:**
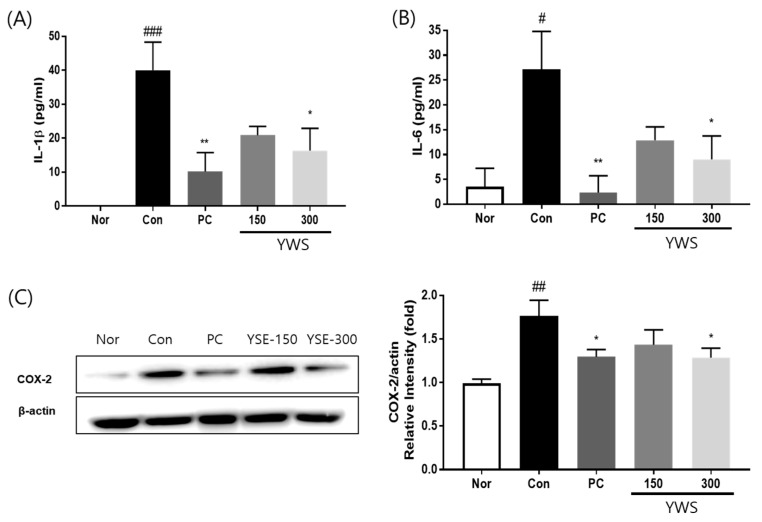
Effects of YWS on the expression of cytokines and inflammatory mediators in a model of EtOH-induced gastric ulcer. The expression of (**A**) IL-1β, (**B**) IL-6, and (**C**) COX-2 was determined using ELISA and WB (n = 8) and Western blot analysis (n = 3). Values are expressed as mean ± SEM. ^#^ *p* < 0.05, ^##^ *p* < 0.01, and ^###^ *p* < 0.001 versus the normal control group (Nor); and * *p* < 0.05, and ** *p* < 0.01, versus the gastritis control group (Con).

ELISA showed that the expression of IL-1β and IL-6 in the serum increased following ethanol-mediated induction of gastric ulcers, and this increase was significantly inhibited by treatment with YWS at 300 mg/kg (Figure 7). YWS at 150 mg/kg also partially suppressed the expression of IL-1β and IL-6, although not as significantly as at 300 mg/kg. Additionally, individual pretreatment with 300 mg/kg YWS and PC significantly reduced the expression of COX-2 (compared with the gastritis control group).

### 2.7. Effect of YWS on Histamine and PGE2 Secretion in a Model of EtOH-Induced Gastric Ulcer

**Figure 8:**
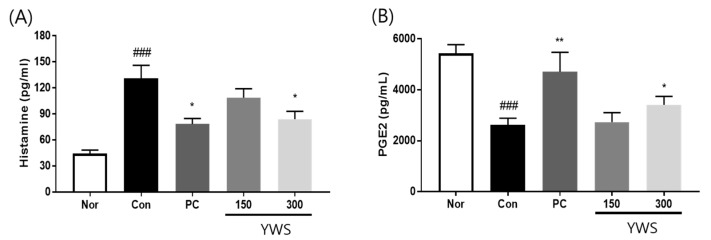
Effects of YWS on the expression of histamine and PGE2 in a model of EtOH-induced gastric ulcer. The expression of (**A**) histamine and (**B**) PGE2 levels were determined using ELISA. Values are expressed as mean ± SEM (n = 8). ^###^ *p* < 0.001 versus the normal control group; and * *p* < 0.05, and ** *p* < 0.01, versus the gastritis control group.

To investigate the changes in gastric secretion-related factors following YWS administration, histamine and PGE2 levels were compared in mice with gastric mucosal damage (Figure 8). PGE2 concentration was significantly increased in EtOH-treated mice administered YWS at a dose of 300 mg/kg and in the PC group. In contrast, plasma histamine levels were significantly reduced in the YWS-treated and PC groups compared to those in the gastritis control group. These results indicate that YWS induces gastric protection by decreasing histamine levels and enhancing PGE2 levels in the gastric mucosa.

## 3. Discussion

The stomach is continually exposed to substances that can cause epithelial damage, resulting in the reduced production of protective substances such as mucus and acidic digestive fluids. Mucosal integrity is maintained by defence factors, including the epithelial barrier, and blood flow regulation, as well as mucus, bicarbonate, prostaglandin, and growth factors. However, when there is an imbalance between protective mechanisms and harmful substances, the protective mechanisms can be weakened and gastric acid secretion increased, thereby damaging the gastric mucosa [13,14]. The gastric acid secretion pathway involves the activation of various receptors expressed in parietal cells. Gastric acid secretion is primarily regulated by the activation of CCK2R by gastrin, H2R by histamine, and M3R by acetylcholine [15,16,17]. The activation of H2R, M3R, and CCK2R results in an increased concentration of cAMP in parietal cells and the activation of H^+^/K^+^ ATPase. In the final step within parietal cells, H^+^/K^+^ ATPase (proton pump) is activated, pumping protons into the stomach, thereby promoting gastric acid production [15,18,19]. PPIs currently used to treat gastritis, such as omeprazole, esomeprazole, and lansoprazole, inhibit the H^+^/K^+^ ATPase located in the parietal cells of the stomach wall [20]. H2 blockers, including ranitidine, famotidine, and cimetidine, block H2R on parietal cells. This inhibition reduces stomach acid production, a phenomenon that helps heal the stomach lining and reduces irritation and inflammation [21]. Therefore, to confirm the protective effects of YWS against gastric mucosal damage related to gastric acid secretion, we utilised an animal model of restraint stress-induced gastritis, which was confirmed through changes in factors related to gastric acid secretion. Additionally, we investigated the anti-inflammatory activity and inhibitory effects of EtOH on gastric mucosal damage in an animal model of acute gastritis. Chronic stress stimulates the secretion of adrenal corticosteroids by the sympathetic nervous system, leading to excessive gastric acid secretion, ultimately resulting in inflammatory damage and gastric ulcers [22]. Stress-induced corticosterone increase lead to the release of gastrin by vagal stimulation and decreases PGE2 levels, resulting in an imbalance in gastric acid secretion levels and the gastric mucosal barrier [23]. In this study, we measured the serum levels of corticosterone, the main glucocorticoid, in a model of restraint stress-induced gastritis to assess the stress response [24,25]. In our study, corticosterone levels were increased by restraint stress, and YWS administration clearly reduced corticosterone release. Furthermore, this study revealed a significant reduction in PGE2 levels along with a marked increase in gastrin levels in the restraint stress-induced gastritis group, which may be ascribed to stress-induced corticosterone [26]. Prostaglandin E plays a crucial role in gastric mucosal defence and is reported to inhibit ulcer formation by regulating various protective actions in gastric cells, including gastric acid secretion, mucin synthesis and secretion, blood flow, and gastric motility [27,28]. This present study shows that YWS remarkably elevated the expression of PGE2 (Figure 4D) and mucin genes (Figure 5A) in stress-induced animals. In addition, YWS inhibited histamine secretion and H2R activation, resulting in a decrease in intracellular cAMP levels in parietal cells, ultimately leading to a decrease in H^+^/K^+^ ATPase levels. However, no inhibitory effect of YWS on gastrin was observed. This was consistent with the finding that YWS does not affect gastrin-induced CCK2R activation. Histamine, released from enterochromaffin-like (ECL) cells in response to stimulation of the gastric mucosa by gastrin and acetylcholine, causes increased secretion of gastric acid during gastric irritation or inflammation and damages the mucous membrane, thereby aggravating gastritis symptoms [29]. Moreover, histamine induces increased acid secretion and mucosal damage during gastric inflammation, which is associated with pro-inflammatory cytokines, including IL-1β. IL-6 has also been identified as a major driver of gastric ulcer development [30,31,32,33]. COX-2 is an inflammatory factor that is mainly expressed in inflamed tissues; it gradually accelerates damage to the gastric mucosa stimulated by aggressive external factors [34,35]. We further studied the potential anti-inflammatory activity of YWS, i.e., reducing both macroscopic and histopathological lesions in the gastric mucosa and decreasing cytokine levels in an animal model of alcohol gastritis. Acute and chronic alcohol intake causes severe damage to the gastric mucosa in both humans and rodents. Alcohol rapidly penetrates the gastric mucosa, causing vascular damage, reducing blood flow, ultimately resulting in tissue necrosis [36]. This alteration is primarily driven by the inflammatory response triggered by ethanol exposure and is characterized by the release of pro-inflammatory cytokines [32]. In this study, we found increased levels of IL-1β, IL-6, COX-2, and histamine in the ethanol-induced control group, whereas the levels of PGE2 were significantly decreased due to its gastroprotective activity. Treatment with YWS and famotidine remarkably suppressed this increase in the levels of pro-inflammatory cytokines, and histamine production enhanced PGE2 levels, demonstrating the anti-inflammatory and gastroprotective potential of YWS (Figure 4 and Figure 8).

*G. uralensis*, an ingredient of YWS, is a medicinal herb that has long been used to treat gastrointestinal diseases in China [11,37]. Furthermore, baicalin, the main ingredient of YWS, and other minor ingredients, such as poncirin, naringin, and wogonin, exert anti-inflammatory and protective effects against ethanol-induced gastric damage [38,39,40,41]. However, the doses of *G. uralensis* and other compounds contained in YWS used to evaluate anti-gastritis efficacy in previous studies were higher than those used in YWS in this study. Considering these reports on the representative components, it can be inferred that the therapeutic effects of YWS could be due to the synergistic anti-inflammatory and gastroprotective effects of its compounds. Further investigation into the effects of other compounds included in YWS and their potential synergistic impact on gastritis is needed.

## 4. Materials and Methods

### 4.1. YWS Extract Preparation

To prepare the YWS extract, six herbal medicines, *P. trifoliata*, *O. gigas*, *G. uralensis*, *S. baicalensis*, Massa. medicata Fermentata, and *P. bambusoides*, were purchased from Kwangmyungdang Medical Herbs (Ulsan, Republic of Korea). *O. gigas* was extracted with water, while the remaining five species were extracted using 30% ethanol (EtOH). Each herbal medicine was extracted using the reflux method, concentrated under reduced pressure, and freeze dried. These extracts were combined in specific proportions (Table 2) to formulate the final YWS extract (*w*/*w*) [12]. Specimens of each herbal medicine in YWS were stored in the Korean Medicine Science Research Division, Korea Institute of Oriental Medicine (KIOM).

### 4.2. Phytochemical Analysis

Freeze-dried YWS samples were analysed using a Prominence LC-20A series HPLC system (Shimadzu Corporation, Kyoto, Japan) with a photodiode array detector (PDA). This HPLC–PDA system was controlled using LabSolution software (Version 5.117, Shimadzu). Chromatographic separation of the 14 target components was performed using a SunFire C_18_ column (Waters, Milford, MA, USA) maintained at 35 °C and a water (solvent A)-acetonitrile (solvent B) mobile phase system (both containing 0.1% formic acid). The mobile phase flowed through the following gradient elution: 20% B (initial time), 60% B (40 min), 95% B (50 min and held for 5 min), and 20% B (60 min and held for 10 min). The PDA scanned the range of 190–400 nm for profiling, with quantification at 250, 275, 280, 310, and 370 nm, respectively. The standard and sample solutions were flowed at a rate of 1.0 mL/min and 10.0 μL of the sample was injected for analysis.

The standard stock solution for HPLC–PDA profiling analysis of target components from the YWS sample was prepared using methanol (1.0 mg/mL) and stored in a refrigerator (approximately 4 °C) until use. The sample solution was prepared using 70% methanol at a concentration of 10 mg/mL. Approximately 100 mg of the lyophilised YWS powder sample was collected, 10 mL of 70% methanol was added, and ultrasonic extraction was performed for 1 h at room temperature. Subsequently, the extract solution was filtered using a 0.22 μm ABLUO^®^ syringe filter (GVS Spa, Bologna, Italy) before injection into the HPLC instrument.

### 4.3. Evaluation of Gastroprotective and Anti-Ulcer Activity

ICR mice (30–35 g) and C57BL/6 mice (20–24 g) were purchased from Orient Bio (Seongnam, Republic of Korea). The animal experiments were approved by the Animal Experimentation Ethics Committee of the Korea Institute of Oriental Medicine (Approval code: 22-084, 23-030).

#### 4.3.1. Ethanol-Induced Gastric Ulcer

ICR mice were randomly assigned (n = 5) to different groups. Normal control (Nor), gastritis control (Con), YWS (150 and 300 mg/kg), and famotidine (positive control (PC), 5 mg/kg) group were orally administered to mice in each group for 10 days. On the sixth day, 50% EtOH was orally administered to animals in each group to induce gastric ulceration over 4 days. On the tenth day, the mice were fasted for 20 h with access to tap water. After 1 h of oral treatment, each animal received 50% EtOH. One hour later, blood samples were collected via cardiac puncture under anesthesia and the mice were sacrificed to remove the gastric portion, which was opened along the greater curvature, washed with saline solution (0.9%), and photographed for analysis. The ulcerated area was quantified using computerized planimetry (ImageJ v1.53t), and the results were expressed as percentages [13,42].

#### 4.3.2. Restraint Stress-Induced Gastric Ulcer

C57BL/6 mice were randomly assigned (n = 8) to different groups. The non-stress control (Nor), stressed control (Con) (0.5% carboxymethyl cellulose [CMC]) and YWS (150 and 300 mg/kg), and famotidine (positive control (PC), 5 mg/kg) groups were orally administered their respective treatments for 23 d. On the eighth day, after 1 h of drug treatment, individual mice were physically restrained in a restraint device (3 cm in diameter) without food or water for 5 h per day for 16 days. Control mice were left in their usual cages for the same duration without food or water [43]. After the final restraint, mice were fasted for 16 h and sacrificed. The stomach was opened along the greater curvature and washed with saline solution. The severity of gastric mucosal injury was determined using the ulceration index (UI) for all mice groups, which was classified as follows: 0 = no lesions (normal stomach); 0.5 = hyperemia; 1 = hemorrhagic spots; 2 = one to five small ulcers; 3 = many small ulcers; 4 = one to five small and one to three large ulcers; 5 = many small and large ulcers; 6 = stomach full of ulcers along with perforations. Percentage protection was calculated in comparison with the non-stress control group [44].

### 4.4. ELISA

Serum samples were obtained from all mice by centrifugation at 3000 rpm for 15 min at 4 °C and then stored at −70 °C until use for ELISA. Gastric mucosa was treated with cAMP assay cell lysis buffer and ground using a tissue homogeniser. The gastric mucosal suspension was centrifuged at 2000× *g*, and the supernatant was used to measure cAMP levels using a cAMP assay kit (Abcam, Boston, MA, USA). The levels of IL-1β, IL-6, PGE2, histamine, gastrin, and corticosterone were measured using ELISA kits procured from R&D Systems, as per the manufacturer’s instructions.

### 4.5. Quantitative RT-PCR

Total RNA was extracted from stomach tissues using an RNeasy Mini Kit (Qiagen, Hilden, Germany), and 1 μg of this RNA was reverse-transcribed into cDNA using an iScript cDNA synthesis kit (Bio-Rad) following the manufacturer’s instructions.

The obtained cDNA was subjected to qPCR on a CFX Connect Real-Time polymerase chain reaction (PCR) System (Bio-Rad, Hercules, CA, USA) using Power SYBR Green PCR Master Mix (Bio-Rad, Hercules, CA, USA), with the following cycling parameters: 10 min initial denaturation at 95 °C, followed by 40 cycles of 10 s at 95 °C and 60 s at 60 °C. Relative gene expression levels were represented as ^Δ^Ct = Ct target gene − Ct housekeeping gene (glyceraldehyde-3-phosphate dehydrogenase; GAPDH). Changes in gene expression were calculated using the 2^−ΔΔCT^ method. Table 3 lists the primer sequences used in this study.

### 4.6. Immunoblot Analysis

Total protein from stomach tissue was collected with RIPA buffer. An amount of 20 μg protein was separated by 10% SDS-PAGE electrophoresis and then transferred onto PVDF membranes (Millipore, Burlington, MA, USA). After blocking with 5% non-fat milk in Tris-buffered saline containing 0.05% Tween-20 for 1 h, membranes were incubated with primary antibodies overnight at 4 °C, followed by HRP-labeled secondary antibodies for 1 h at room temperature. After washing with TBST, the enhanced chemiluminescence (ECL) solution was added. β-actin was used as the internal reference, and the relative expression was quantified using ImageJ.

### 4.7. Histological Analysis

Stomach tissues were collected for histological analysis and fixed in 10% neutral buffered formalin (NBF, Sigma, Burlington, MA, USA). The fixed samples were embedded in paraffin and sectioned (5 µm sections). The tissue sections were stained with haematoxylin and eosin (H&E) and periodic acid-Schiff (PAS).

## 5. Conclusions

These results suggest that YWS exerts protective effects on the gastric mucosa and significantly downregulates inflammatory cytokines and COX-2. YWS inhibited histamine secretion, prevented H2R activation, and reduced cAMP levels, consequently decreasing the activity of H^+^/K^+^ ATPase, resulting in reduced gastric acid secretion. Additionally, YWS significantly increased the secretion of protective factors, such as PGE2 and mucin, confirming its potential for gastric mucosal protection. Our results suggest that YWS may be an effective candidate for the treatment of gastritis.

## Figures and Tables

**Table 1:** Amount of the 14 compounds in the freeze-dried YWS sample (*n* = 5).

Compound	Mean (mg/g)	SD	RSD (%)
Liquiritin apioside	6.93	0.04	0.63
Liquiritin	4.13	0.04	0.95
4-Hydroxycinnamic acid	0.64	0.02 × 10^−1^	0.24
Narirutin	2.56	0.04 × 10^−1^	0.15
Naringin	18.44	0.04	0.22
Ononin	1.45	0.01	0.51
Baicalin	89.45	0.25	0.27
Neoponcirin	4.98	0.01	0.13
Poncirin	31.81	0.08	0.26
Wogonoside	15.29	0.01	0.08
Baicalein	2.62	0.03	1.32
Isoliquiritgenin	0.03	0.03 × 10^−2^	1.16
Glycyrrhizin	19.48	0.02	0.08
Wogonin	0.92	0.02 × 10^−1^	0.19

**Table 2:** Yield and mixing portion of herbal medicines comprising Yeokwisan (YWS).

Herbal Medicines	Extraction Solvent	Yield (%)	Mixture Portion (%)
*G*. *uralensis* Fisch.	30% EtOH	24.7	16.9
Massa Medicata Fermentata	30% EtOH	9.3	8.4
*P*. *bambusoides* Sieb. et Zucc.	30% EtOH	5.6	5.0
*P*. *trifoliata* (L.) Raf.	30% EtOH	19.4	17.5
*S*. *baicalensis* Georgi	30% EtOH	50.2	45.4
*O*. *gigas* Thunb	100% H_2_O	1.9	6.7

**Table 3:** Primer sequences used for the mRNA expression analyses.

Gene		Primer Sequence
*MUC5AC*	Forward	5′-CCTCTCAGAGGAATGTGACTCTGCGC-3′
	Reverse	5′-CCAGGCAGCCACACTTCTCAACCT-3′
*H2R*	Forward	5′-CCTTCTCTGCCATTTACCAGTTG-3′
	Reverse	5′-CATCACATCCAGGCTGGTGTAG-3′
*CCK2R*	Forward	5′-GATGGCTGCTACGTGCAACT-3′
	Reverse	5′-CGCACCACCCGCTTCTTAG-3′
*M3R*	Forward	5′-AAGGCACCAAACGCTCATCT-3′
	Reverse	5′-GCAAACCTCTTAGCCAGCGT-3′
*H^+^/K^+^ ATPase*	Forward	5′-ATGGCCCGCGGAAAAGCCAAGGAGGAAGGCA-3′
	Reverse	5′-GATCAGCTCTTAATTTCAATTTTTCATCAAAG-3′
*GAPDH*	Forward	5′-CATACCAGGAAATGAGCTTG-3′
	Reverse	5′-ATGACATCAAGAAGGTGGTG-3′

## Data Availability

The original contributions presented in the study are included in the article, further inquiries can be directed to the corresponding authors.
